# Lupus Vulgaris

**Published:** 1886-01

**Authors:** Henry J. Reynolds

**Affiliations:** Professor of Skin-Diseases, Dermatologist to the West-Side Dispensary, Surgeon to the Department of Genito-Urinary Diseases, West-Side Dispensary, Chicago


### CLINICAL REPORTS.

CLINICAL LECTURE ON LUPUS VULGARIS, AT THE COLLEGE OF PHYSICANS AND SURGEONS OF CHICAGO. *By* HENRY J. REYNOLDS, M. D. *Professor of Skin-Diseases, Dermatologist to the West-Side Dispensary, Surgeon to the Department of Genito-Urinary Diseases, West-Side Dispensary, Chicago.*

*Gentlemen:*

I wish to invite your attention to-day to a consideration of the disease known as lupus vulgaris.

The case of the lady before us illustrates the disease in a most typical manner, but before referring to this individual case, I prefer that you have some knowledge of the general characteristics of the disease.

Lupus vulgaris belongs to the class of skin diseases known as "new growths," and owing to the varied appearance of which, in different cases and at different times, numerous names have been applied to it; as lupus exedens, lupus verrucasus, lupus discretus, lupus vegetans, etc., all of which, however, are embraced under the head of lupus vulgaris, and are simply different manifestations of the same disease. It must be understood, however, that the name does not embrace the disease known as lupus erythematosus, which, though somewhat similar in behavior, is usually regarded as an entirely distinct affection. Lupus vulgaris generally first manifests itself in childhood. If not seen by the physician till adult life or old age, the previous history—old scars, etc.—will almost invariably reveal the previous existence of the disease or of trouble of a similar sort. It may last all through life, and it is not contagious. It may occur upon any portion of the integument of the body or of the contiguous mucous membrane, but it is most frequently found upon the face, with a preference for the nose. In the destructive process the skin, mucous membrane and cartilages may be involved, but unlike syphilis it spares the bone. The disease first manifests itself in the form of soft pin-head to small pea-sized brownish red or yellowish papules which, from being deeply seated in the corium, are at first scarcely perceptible to the touch. By enlargement and aggregation of these small growths, which progress extremely slowly, larger nodular or tubercular masses are formed; and by the subsequent breaking down of the same, we have the characteristic ulceration, cicatrization. etc. The small growths may, on the contrary, atrophy and become absorbed without breaking down. The disease, though chronic and very slow in its progress, is an extremely destructive one, especially in the region of the face, sometimes destroying almost the entire nose, eyelids, etc. There is an entire lack of symmetry in the disposition of the lupous growth, occurring as it does in irregular, uneven patches here and there. It may involve only one side of the face and at the same time a portion of an extremity, as in a case at present under my care. The disease, though not uncommon in Germany, is quite rare in this country. The ulcer of lupus vulgaris has a reddish, indolent, granulating base which bleeds easily, and is surrounded by a soft, flat, neither elevated nor undermined margin. Crusting is not a prominent feature, and there is little or no pain connected with the disease.

DIAGNOSIS.—The principal diseases from which lupus vulgaris is to be differentiated are syphilis, superficial epithelioma and lupus erythematosus. By close attention to the following diagnostic points, a diagnosis can almost invariably be made with positiveness :

| LUPUS VULGARIS. | SYPHILIS. |
| --- | --- |
| Almost invariably first manifests itself in childhood. | Usually in adult life. |
| Is not hereditary. | May be. |
| Never occurs in infancy. | It may. |
| Rare in this country. | More common. |
| Papules small, soft, deep, not elevated, and may reappear in the scar. | Larger, harder, elevated, and never reappears in the scar. |
| If female, not necessarily any previous history of abortions. | If female, may have previous history of abortions. |
| Generally local. | Liable to be a tendency to general distribution. |
| Progresses very slowly. | More rapid. |
| Margin ill-defined and uneven. | Well defined. |
| Never affects the bone. | It may. |
| No syphilitic history. | Almost invariably a history of primary and secondary syphilis in adult. |

THE ULCER.

| | |
| --- | --- |
| Generally begins from an aggregation of points or papules. | Generally from single point. |
| Is shallow, with red granulating base. | Deep. |
| Margin not abrupt, elevated nor undermined. | Margin, abrupt, elevated and undermined. |
| Secretion scanty. | More profuse. |
| Odorless. | Fetid. |
| Little crusting. | More crusting. |
| Resulting scar always white. | Often pigmented. |

| LUPUS VULGARIS. | SUPERFICIAL EPITHELIOMA. |
| --- | --- |
| Generally begins in childhood. | Usually found in old age. |
| Starts as multiple discrete papules which may each form an ulcer. | Starts within from one or an aggregation of papules which ultimately form only one ulcer. |

| | |
|---|---|
| Not painful. | More or less painful. |
| Progresses very slowly. | Progresses more rapidly. |
| Becomes irregularly nodular. | Does not become nodular. |
| Becomes diffuse and ill-defined. | Does not become diffuse and is well defined. |

THE ULCER.

| | |
|---|---|
| Generally multiple. | Usually single. |
| Shallow, with red granulating base which bleeds easily. | Deep, with hard indurated, uneven base. |
| Edges ill-defined and neither elevated, everted, nor undermined. | Edges clean-cut, abrupt, indurated, and everted. |
| Ill-defined, red, unhealthy skin outside the margin of the ulcer, due to the more diffuse nature of the disease. | Surrounded by healthy skin. |
| Moderate amount of secretion. | Secretion more scanty and liable to be tinged with blood. |
| Secretion inodorous. | Secretion fetid. |
| Presence of the characteristic non-ulcerative papules found in the scar and healthy skin. | No elementary papules found at same time as ulcer. |

Lupus erythematosus may at once be differentiated from lupus vulgaris by the absence in the former of deep-seated papules, and of ulceration and discharge by its occurring first in adult life, by its symmetrical distribution on each side of the face, as a rule, by its firmly adherent yellow scales, and by the absence of the nodular condition characteristic of lupus vulgaris.

The history of the case we are now about to operate upon is as follows: She is aet. 48, German, though thirty years in this country. Always enjoyed good health (except that she was always subject to what was called eczema) until five years ago, at which time her present disease broke out, appearing first on the right leg, below the knee, where you now see the scar, followed very shortly afterwards by a similar outbreak in several places on the face. The disease gradually grew worse ever since, some patches becoming atrophied and absorbed and others going on to ulceration, and subsequently cicatrization, etc., till we now find the present distorted, ulcerated condition. You will notice that the lower eye-lid is all gone, both alæ of the nose, and the upper lip are badly involved though not yet ulcerated; an ulcer the size of a dollar on the centre of the fore-head and an indurated, red, nodular and partly ulcerated surface one to two inches in diameter, extending from just below the left eye downwards past the left angle of the mouth to a point beneath the chin.

TREATMENT.—While the patient is being placed under ether we will refer briefly to the treatment. Various methods and remedies are adopted in the treatment of this disease; the simplest, and I may say, the least effective, being the local application of such remedies as green soap, stimulating lotions, caustics, etc. When the affection is mild and there is already a tendency to atrophy and absorption such treatment may be all that is required. Another and more effectual method is that of multiple linear scarification of the part; a number of parallel incisions being made just deep enough to go down to the healthy tissue, the same being then crossed by similar incisions. Another, the most effectual in advanced cases, and the one we will adopt in this case is that of erasion or scraping with the dermal curette. We will now proceed with the operation. The spoon-shaped instrument I now show you is what is called the dermal curette and with it we will scrape away as far as possible the diseased tissue, which as you will observe is easily done, as this lupus growth, whether papular, tubercular or advanced to ulceration, gives way very readily in front of the curette, and scrapes out almost like cheese or rotten wood, leaving at the margins the normal tissue, which is much more resisting and cannot readily be thus removed. You will observe also that more of this tissue scrapes away than we would have expected, but we must go on till we meet with the resisting healthy tissue. You see we have been obliged, with the rest, to remove almost completely the alæ of the nose, however, in advanced cases like this, nothing short of heroic measures would be of any avail. Having now gotten rid of everything that can be scraped away, we will apply nitrate of silver thoroughly to all the scraped surface, as also to the eyelid and inside of the nose, with a view to destroying any remaining diseased tissues; and will order, as after-treatment, an ointment of iodoform ʒ i, pyrogallic acid ʒ i, Ung. aq. Rosæ ℥ i.

One peculiarity of this case is that we have not good evidence of the existence of the disease in early life. It is not known however, that she did *not* have it, as a history is given of "eczema" which may in reality have been lupus; the papules constantly becoming atrophied and absorbed without giving rise to any well marked lupus symptoms until later in life.*

---

### MERCY HOSPITAL SURGICAL CLINIC, OF PROFESSOR EDMUND ANDREWS, M. D., LL. D.

### I. FISTULA OF THE NECK.

This patient has a *fistula* in his neck, just below and behind the angle of the inferior maxillary bone. Some of the glands in this region are enlarged, but I cannot say whether it is from tubercular infiltration or some other cause, either malignant or

*The patches were entirely healed in three weeks after the operation, but a few spots will require to be scraped again.

